# Psychiatric consequences of a father’s leave policy by nativity: a quasi-experimental study in Sweden

**DOI:** 10.1136/jech-2021-217980

**Published:** 2021-10-11

**Authors:** Helena Honkaniemi, Srinivasa Vittal Katikireddi, Mikael Rostila, Sol P Juárez

**Affiliations:** 1 Department of Public Health Sciences, Stockholm University, Stockholm, Sweden; 2 Centre for Health Equity Studies (CHESS), Stockholm University/Karolinska Institutet, Stockholm, Sweden; 3 MRC/CSO Social and Public Health Sciences Unit, University of Glasgow, Glasgow, UK

**Keywords:** human migration, psychiatry, policy

## Abstract

**Background:**

Parental leave use has been found to promote maternal and child health, with limited evidence of mental health impacts on fathers. How these effects vary for minority populations with poorer mental health and lower leave uptake, such as migrants, remains under-investigated. This study assessed the effects of a Swedish policy to encourage fathers’ leave, the 1995 *Father’s quota*, on Swedish-born and migrant fathers’ psychiatric hospitalisations.

**Methods:**

We conducted an interrupted time series analysis using Swedish total population register data for first-time fathers of children born before (1992–1994) and after (1995–1997) the reform (n=198 589). Swedish-born and migrant fathers’ 3-year psychiatric hospitalisation rates were modelled using segmented negative binomial regression, adjusting for seasonality and autocorrelation, with stratified analyses by region of origin, duration of residence, and partners’ nativity.

**Results:**

From immediately pre-reform to post-reform, the proportion of fathers using parental leave increased from 63.6% to 86.4% of native-born and 37.1% to 51.2% of migrants. Swedish-born fathers exhibited no changes in psychiatric hospitalisation rates post-reform, whereas migrants showed 36% decreased rates (incidence rate ratio (IRR) 0.64, 95% CI 0.47 to 0.86). Migrants from regions not predominantly consisting of Organisation for Economic Cooperation and Development countries (IRR 0.50, 95% CI 0.19 to 1.33), and those with migrant partners (IRR 0.23, 95% CI 0.14 to 0.38), experienced the greatest decreases in psychiatric hospitalisation rates.

**Conclusion:**

The findings of this study suggest that policies oriented towards promoting father’s use of parental leave may help to reduce native–migrant health inequalities, with broader benefits for family well-being and child development.

## Introduction

Parental leave policies have been shown to promote maternal and child health,^1^ yet the influence of fathers’ leave for their own health remains understudied. Around 10% of fathers worldwide suffer from depression in the postpartum period,^2 3^ alongside heightened levels of stress and anxiety.^4^ Longer fathers’ leave could protect their mental health through biological (ie, hormonal) changes^5^; psychosocial changes, by fostering work–family balance^6^ and family relationships^7 8^; and health behavioural changes, by encouraging child-friendly behaviours such as physical activity^9^ and decreased alcohol consumption.^10 11^ However, little is known about the mental health consequences of fathers’ leave, and whether these consequences differ for minority groups such as migrants, who experience worse mental health^12^ and lower parental leave uptake^13 14^ than natives.

Sweden has one of the most generous parental leave systems worldwide, making it an apt target for investigating these health effects. Today, residents qualify for 480 days of job-protected leave per child (240 days per parent), of which 390 are paid at a low flat rate for unemployed and low-earning parents or 80% of higher earnings, and 90 at a universal flat rate. Although fathers have been eligible to use parental leave since 1974, most days continue to be claimed by mothers.^13^ In response, the *Father’s quota* was implemented in 1995, reserving a month of leave (to be forfeited if left unused) to fathers of children born from 1 January 1995, and successfully increasing the proportion of fathers using leave from 43% to 75%.^15^

Meanwhile, migrant fathers in Sweden have used even less leave than natives, in part due to greater levels of unemployment and low income (ie, being more likely to receive flat-rate benefits); limited institutional knowledge (ie, to apply for parental leave benefits); and conflicting cultural and gender norms (ie, being more inclined to remain at work than home for childcare).^13^ These factors vary across migrant groups, including by region of origin, duration of residence and partners’ nativity, indicative of integration levels in the receiving country.^16 17^ Promoting migrant fathers’ leave use, especially among less integrated migrants, could contribute to narrowing native–migrant mental health inequalities.^12^

Given the possibility of confounding and health selection, quasi-experimental designs are useful to isolate causal effects between parental leave and health.^18^ One quasi-experimental study found decreased sick leave uptake among fathers following a Norwegian father’s quota reform,^19^ but no such evidence exists in Sweden. Furthermore, no study has considered the differential effects of these policies on marginalised groups such as migrants. This study thus aims to assess the effects of the 1995 *Father’s quota* on Swedish-born and migrant fathers’ psychiatric hospitalisations, further exploring variation by migrants’ integration.

## Methods

### Data sources and study population

The study was conducted as part of the Unintended health consequences of Swedish parental leave policy (ParLeHealth) project, based on a pre-published study protocol.^20^ In a quasi-experimental design, the 1995 *Father’s quota* was used as an exogenous intervention to incentivise fathers’ leave uptake.^18^ Longitudinal total population data were drawn from linked Swedish registers, including the Total Population Register (TPR) and Multigenerational Register (MGR); the Longitudinal Integration Database for Health Insurance and Labour Market Studies (LISA); and the Medical Birth Register (MBR), National Patient Register (NPR) and Cause of Death Register (CDR).

**Figure 1:**
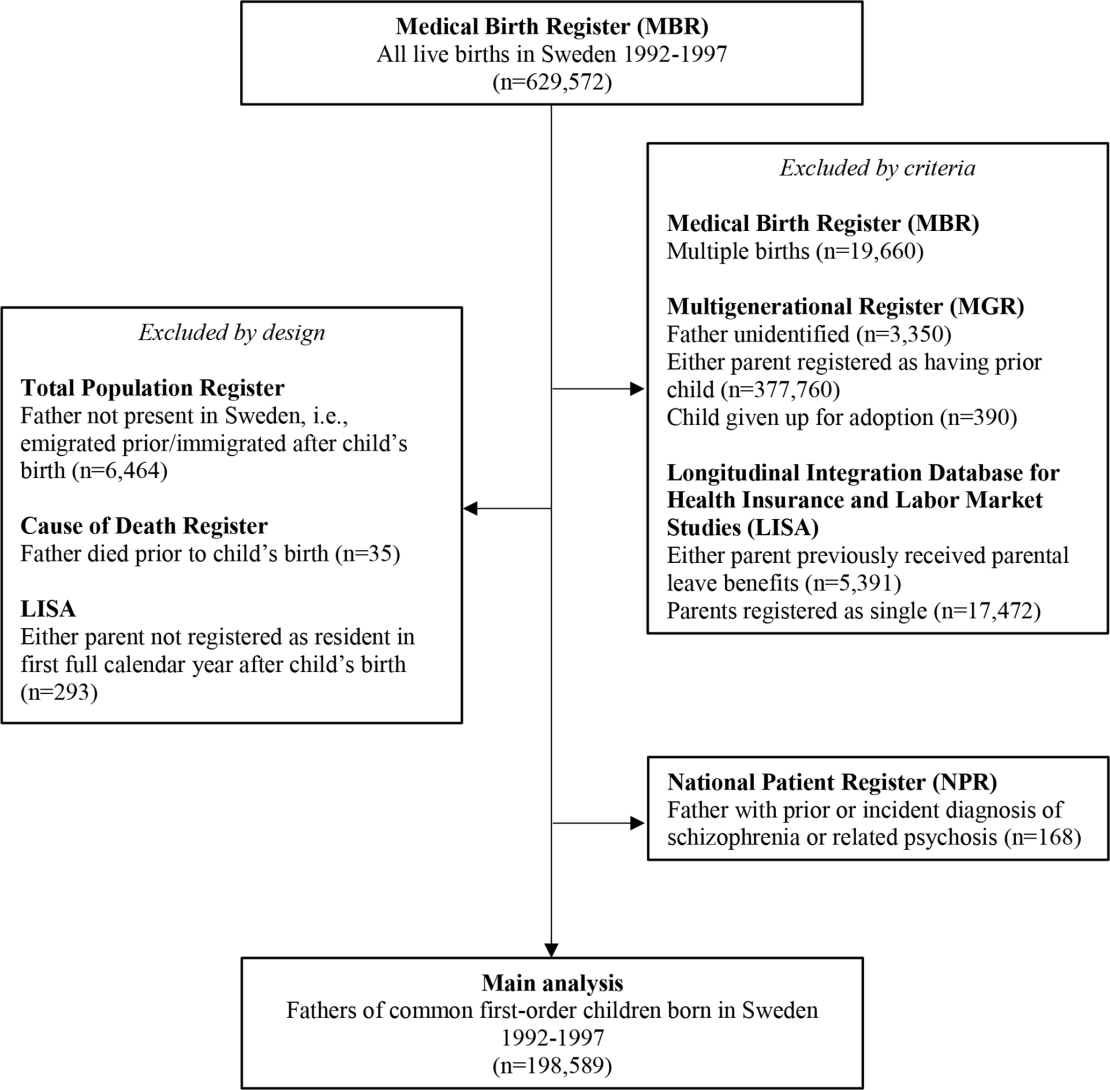
Flowchart for sample selection: First-time fathers of singleton children born in Sweden, 1992–1997 (n=198 589).

We included first-time fathers with singleton children born in Sweden 3 years before (1992–1994) and after (1995–1997) the 1 January 1995 implementation of the *Father’s quota* (figure 1). Using the MBR, we identified all live births from 1 January 1992 to 31 December 1997 (n=629 572), excluding multiple births (n=19 660). Maternal and child records were then linked to fathers’ data, excluding family units with unidentifiable fathers (MGR; n=3350). We focused on common biological first-order children, given that we could not distinguish leave use for multiple children^21^ and that parents were less likely to adjust their uptake behaviours if they had used parental leave prior to the reform (ie, contamination of the policy effect).^13^ Parents coded as having previous children (MGR; n=377 760) or as having taken out parental leave prior to the study period (LISA; n=5391) were thus excluded. Given that parental leave benefits are contingent on custody,^13^ parents of children given up for adoption (MGR; n=390) and those coded as single (ie, not cohabiting or married) in the year of childbirth (LISA; n=17 472) were also excluded. We then removed fathers that had emigrated from or not yet migrated to Sweden (TPR; n=6464) or died (CDR; n=35) by their child’s birth. Missing socioeconomic data in the first full calendar year after birth was also used to exclude non-residents (LISA; n=293). Finally, we excluded fathers with previous or incident hospitalisations for schizophrenia (NPR; n=168), who may have been influenced by a 1995 psychiatric deinstitutionalisation reform which disproportionately affected patients with schizophrenic diagnoses.^22^ The final sample of fathers (n=198 589) was right-censored if they, their partners or children emigrated, died or no longer cohabited prior to the end of follow-up. Observations were aggregated into 72 monthly time points over 6 years of birth data (1992–1997) and 3 years of follow-up (1992–2000).

### Variables

We retrieved data on fathers’ hospitalisation dates and diagnostic codes (International Classification of Diseases, 9^th^ Revision, ICD-9; 10^th^ Revision, ICD-10; NPR). Inpatient hospitalisations with a primary diagnosis of mental and behavioural disorders (ICD-9 290–319; ICD-10 F00–F99), exempting diagnoses of schizophrenia-related disorders (ICD-9 295, 297–298; ICD-10 F20–F29), were counted from the child’s birth month until 36 months postpartum or the end of follow-up. Incident psychiatric hospitalisation events (maximum one daily, irrespective of duration) were presented as rates per 1000 person-years (PY). Although most fathers use leave within 2 years postpartum,^23^ we examined 3-year outcomes to capture potential lagged health effects. For our sensitivity analyses, we estimated a dichotomous measure of fathers’ pre-birth psychiatric hospitalisations (up to 2 years before child’s birthdate) to assess potential health selection; and psychiatric hospitalisation rates up to 18 months postpartum, to exclude potential effects of uncaptured second births.

Fathers were stratified by nativity (ie, Swedish-born or migrant; TPR). Migrants were further stratified by region of origin, including regions predominantly composed of Organisation for Economic Cooperation and Development (OECD) countries (ie, Europe, North America, Oceania) and non-OECD regions (ie, Africa, Asia, South America, stateless/unspecified)^16^; duration of residence (ie, <or≥5 years, first-time migrants only); and partners’ nativity (ie, Swedish-born or migrant).^13^ From LISA, we drew data on annual labour income (continuous; year before childbirth) to assess compositional changes among parents due to the 1990s Swedish financial crisis.^24^ For descriptive purposes, we determined fathers’ unemployment status (whether any labour income was reported; year before childbirth), educational attainment (low: up to 2 years of upper secondary education, medium: up to 2 years of university college, and high: graduate/postgraduate studies; first record in the follow-up period, with multiple imputation for missing values), age (mean; year of childbirth) and parental leave use (whether parental leave benefits were received; up to 3 years after childbirth).

### Analysis

We used interrupted time series (ITS) analysis, which involves plotting a series of observations (fathers’ psychiatric hospitalisation rates) over a period of time ‘interrupted’ by an intervention (the 1995 *Father’s quota*), then fitting and comparing separate regression models for the pre-intervention and post-intervention periods.^25^ We chose this approach due to the availability of a clear intervention date and longitudinal data to establish trends, as well as the possibility to investigate the overall effect of the policy by nativity. Given that the quota was introduced 6 months prior to implementation,^26^ we assumed that fathers could not self-select into reform periods, but were aware of the reform in advance. As such, we expect there to be an immediate stepwise change in hospitalisations due to a rapid response in uptake from 1 January 1995, followed by a gradual slope change as fathers adjust their uptake behaviours over time.^27^ To confirm, we first assessed changes in key covariates by plotting their trends over time, and then specified a segmented negative binomial regression model for psychiatric hospitalisations:

$$\begin{array}{c} \log E\left(Y_{t}\right)=log\left(C_{t}\right)+\beta_{0}+\beta 1T_{t}+\beta_{2}X_{t}+\beta_{3}X_{t}T_{t}+\beta_{4}sin2\pi /12+\beta_{5}cos2\pi /12+\varepsilon_{t} \end{array}$$

Where Y_t_ is the psychiatric hospitalisation count up to 36 months postpartum for fathers of children born in month *t*; log(C_t_) is the logged offset; β_0_ is the baseline rate; β_1_ is the pre-intervention slope, with T_t_ indicating time since the beginning of the study until time *t*; β_2_ is the stepwise change from the observed rate immediately after the intervention compared with the expected counterfactual (with pre-intervention slope), with X_t_ indicating the intervention period; β_3_ is the pre-intervention to post-intervention slope change, with X_t_T_t_ indicating an interaction between intervention period and time; and the sine and cosine terms are Fourier terms adjusting for seasonality.^25 28^ Estimates were presented as incidence rate ratios (IRRs). Post-estimation commands were used to calculate observed and expected rates for fathers of children born in January 1995, absolute rate differences (incidence rate differences) between these rates, and post-reform slopes.^29^ We checked (partial) autocorrelation plots^25^ and calculated 95% CIs using robust or lag-specific Newey-West SE if autocorrelation was present.^28^

Analyses were stratified by nativity, then migrants’ region of origin, duration of residence and partners’ nativity to examine potential effect-measure modification. We conducted sensitivity analyses adjusted for pre-birth psychiatric hospitalisations and annual labour income to address compositional changes in first-time fathers’ health and social characteristics, respectively. Events were restricted to 18 months postpartum to account for the possible influence of an unobserved second birth. Finally, we examined potential confounding from the Swedish financial crisis by specifying a pseudo-intervention date on 1 January 1994. Analyses were conducted in Stata V.15.1.

## Results

Migrant fathers (n=32 868) were on average older, more low-educated or high-educated and lower earning than Swedish-born fathers (n=165 721) (see table 1 for descriptives, online supplemental figures S1-8 for plotted trends). Post-reform first-time fathers were on average older, more educated and more likely to be unemployed or low-earning than pre-reform, with greater changes among migrants than Swedish-born. Migrant fathers were also more likely to be of OECD-origin, with migrant partners and longer residency in Sweden. Migrants were more likely to have pre-birth psychiatric hospitalisations than Swedish-born fathers, although with no change from pre-reform to post-reform. For births before (December 1994) and after (January 1995) the reform, the proportion of fathers using parental leave increased from 63.6% to 86.4% (Swedish-born) and from 37.1% to 51.2% (migrants), differing across migrant subgroups.

10.1136/jech-2021-217980.supp1 Supplementary data

**Table 1:** Descriptive characteristics of first-time fathers of singleton children born from 1992 to 1997, Sweden (n=198 589)

	Swedish-born fathers (n=165 721)		Migrant fathers (n=32 868)	
Children’s birth year	1992–1994	1995–1997	1992–1994	1995–1997
Fathers’ characteristics				
Age* (mean)	29.08	29.66	31.09	31.45
Education† (%)				
Low	13.15	11.61	24.35	22.30
Medium	68.77	69.75	50.14	49.28
High	18.08	18.63	25.51	28.42
Unemployed‡ (% no annual labour income)	4.27	5.26	30.26	40.70
Annual labour income‡ (mean; in thousands, SEK)	161.96	180.51	115.40	126.16
Parental leave use (% receiving parental leave benefits, 0–36 months after birth)	64.67	83.02	37.84	50.24
Migrant fathers’ characteristics				
Region of origin*,§ (%)				
OECD-predominant			51.36	52.94
Non-OECD-predominant			48.64	47.06
Duration of residence*,¶ (%)				
<5 years			37.99	33.20
≥5 years			38.22	39.48
Other (ie, multiple migrations)			23.80	27.32
Partners’ nativity* (%)				
Swedish-born			33.94	30.46
Migrant (foreign-born)			66.06	69.54
Fathers’ psychiatric health				
Pre-birth hospitalisations (% hospitalised, 0–24 months before birth)	0.24	0.24	0.38	0.40
Post-birth hospitalisations (per 1000 person-years, 0–36 months after birth)	2.85	2.50	5.17	3.10

*Measured in child’s birth year.

†First recorded education within the follow-up period; missing values entered with multiple imputation.

‡Measured in the calendar year prior to childbirth.

§OECD-predominant regions consist of Europe, North America and Oceania. Non-OECD-predominant regions are defined as regions with predominantly non-OECD countries, including Africa, Asia, South America and stateless/unspecified origins.

¶Calculated only for first-time migrants to Sweden.

OECD, Organisation for Economic Cooperation and Development; SEK, Swedish kronor.

**Figure 2:**
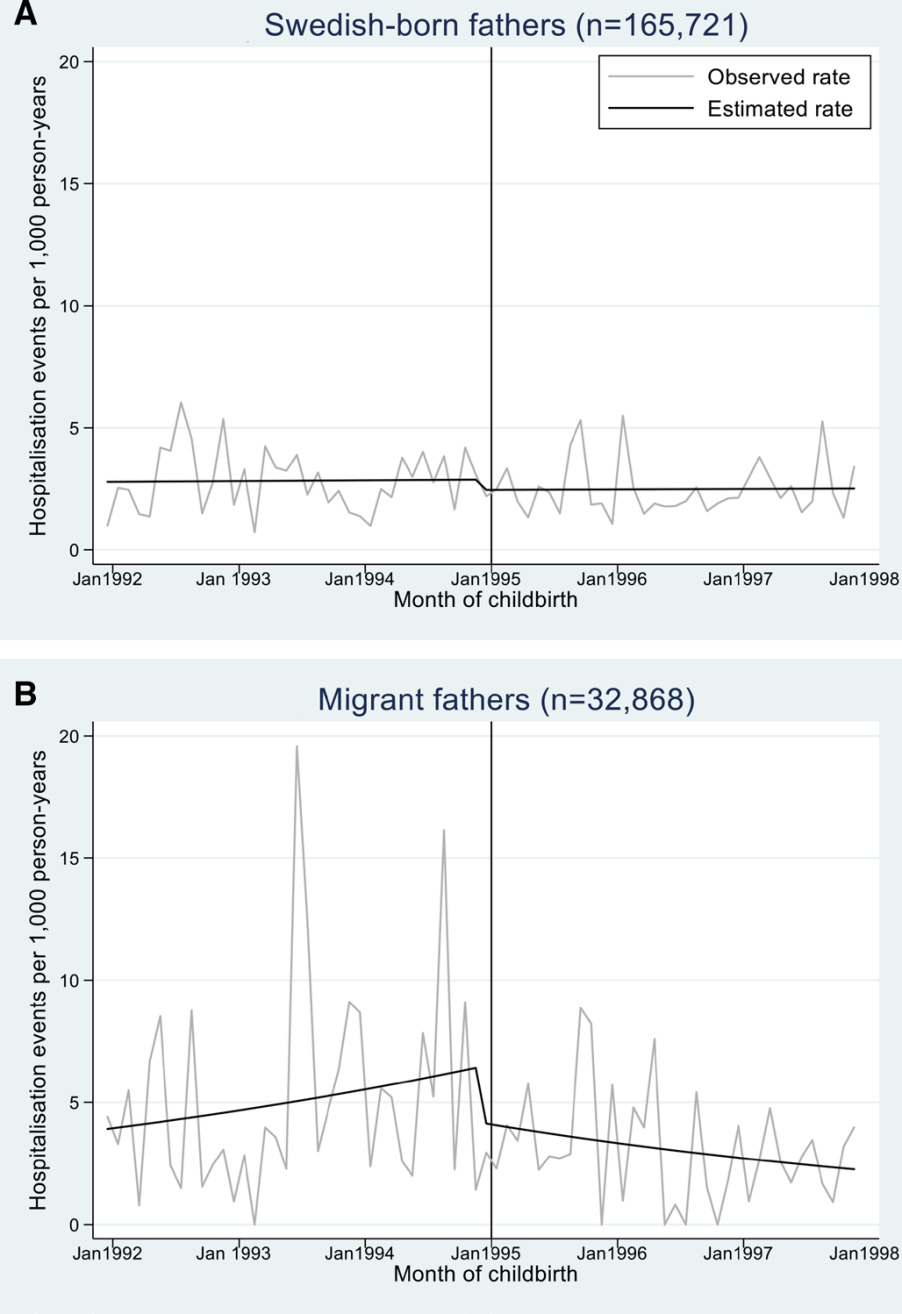
Monthly time-series plots of first-time fathers’ psychiatric hospitalisation rates 0–36 months after child’s birthdate, pooled by child’s birth month (January 1992–December 1997), interrupted by *Father’s quota* (January 1995). Observed rate is the unadjusted hospitalisation rate of fathers by child’s birthdate (monthly data), pooled across the first 36 months after birth. Estimated rate is the adjusted average hospitalisation rate estimated from the fully-adjusted negative binomial regression model with 12-month moving average filters to de-seasonalise the rate.

Swedish-born fathers experienced no stepwise or slope changes in psychiatric hospitalisation rates from before to after the 1995 *Father’s quota*, while migrant fathers experienced a stepwise decrease of 2.39 hospitalisations per 1000 PY (IRR 0.64, 95% CI 0.47 to 0.86), and a reduced slope for every subsequent month of childbirth (0.97, 95% CI 0.95 to 0.99; table 2, figure 2). In subgroup analyses, OECD-origin fathers had a stepwise decrease of 1.87 hospitalisations per 1000 PY (0.66, 95% CI 0.42 to 1.03) but no slope change, while non-OECD-origin fathers experienced a stepwise decrease of 4.60 events per 1000 PY (0.50, 95% CI 0.19 to 1.33) and a slope decrease (0.94, 95% CI 0.90 to 0.98; table 2) (online supplemental figure S9). Short-term residents experienced stepwise decreases of 2.95 hospitalisations per 1000 PY (0.41, 95% CI 0.13 to 1.23), but no slope change post-reform, whereas long-term residents exhibited no stepwise change but a slope decrease (0.93, 95% CI 0.90 to 0.95; table 2) (online supplemental figure S10). Finally, migrant fathers with migrant partners had 5.87 fewer hospitalisations per 1000 PY post-reform (0.23, 95% CI 0.14 to 0.38), without a slope change, whereas those with Swedish-born partners had no stepwise changes but a slope decrease (0.95, 95% CI 0.90 to 0.99; table 2) (online supplemental figure S11).

**Table 2:** First-time fathers’ psychiatric hospitalisation rates 0–36 months after child’s birthdate, pooled by birthdate before (1992–1994) and after (1995–1997) the 1995 *Father’s quota*, Sweden (n=198 589)

	Sample	Baseline*		Rates†								Slopes‡					
				*Expected*		*Observed*		*Stepwise (rate) change*				*Pre-reform*		*Post-reform*		*Slope change*	
	n	Rate	95% CI	Rate	95% CI	Rate	95% CI	IRD	95% CI	IRR	95% CI	IRR	95% CI	IRR	95% CI	IRR	95% CI
Swedish-born	165 721	2.79	2.04 to 3.80	2.90	2.15 to 3.64	2.47	1.83 to 3.11	–0.42	–1.44 to 0.59	0.85	0.59 to 1.24	1.00	0.99 to 1.01	1.00	0.99 to 1.01	1.00	0.98 to 1.02
Migrant	32 868	3.91	2.73 to 5.60	6.55	4.89 to 8.21	4.16	3.24 to 5.08	–2.39	–4.12 to –0.66	0.64	0.47 to 0.86	1.01	1.00 to 1.03	0.98	0.97 to 0.99	0.97	0.95 to 0.99
By region of origin																	
OECD	17 132	5.89	4.11 to 8.44	5.46	2.35 to 8.57	3.60	2.16 to 5.04	–1.87	–4.34 to 0.60	0.66	0.42 to 1.03	1.00	0.98 to 1.02	0.99	0.97 to 1.01	0.99	0.96 to 1.03
Non-OECD	15 736	2.11	1.00 to 4.44	9.15	2.11 to 16.18	4.55	2.47 to 6.63	–4.60	–12.39 to 3.19	0.50	0.19 to 1.33	1.04	1.00 to 1.08	0.98	0.96 to 1.00	0.94	0.90 to 0.98
By duration of residence																	
<5 years	11 727	3.63	2.05 to 6.43	4.97	1.25 to 8.68	2.01	0.44 to 3.59	–2.95	–7.05 to 1.15	0.41	0.13 to 1.23	1.01	0.98 to 1.04	1.01	0.97 to 1.05	1.00	0.96 to 1.05
≥5 years	12 693	3.61	2.13 to 6.14	12.61	9.33 to 15.90	12.26	8.14 to 16.37	–0.36	–5.58 to 4.86	0.97	0.64 to 1.48	1.03	1.01 to 1.06	0.96	0.94 to 0.98	0.93	0.90 to 0.95
By partners’ nativity																	
Migrant partner	22 265	3.11	1.90 to 5.09	7.62	4.63 to 10.61	1.75	1.13 to 2.36	5.87	–8.89 to –2.85	0.23	0.14 to 0.38	1.02	1.00 to 1.05	1.01	0.99 to 1.03	0.99	0.96 to 1.01
Swedish-born partner	10 603	4.33	1.85 to 10.12	7.02	2.13 to 11.90	10.45	5.00 to 15.90	3.43	–4.12 to 10.99	1.49	0.60 to 3.65	1.01	0.97 to 1.05	0.96	0.93 to 0.99	0.95	0.90 to 0.99

Rates are expressed as hospitalisation events per 1000 person-years. CIs are calculated with Huber-White-Sandwich SE, unless autocorrelation is present, when calculated using lag-adjusted Newey-West SE. All models are adjusted for seasonality.

*Baseline rate corresponds to adjusted rate for fathers of children born in January 1992 (*main regression model*).

†Expected rate corresponds to adjusted rate for fathers of children born in January 1995, had the pre-reform slope continued uninterrupted (*post-estimation*). Observed rate corresponds to adjusted rate for fathers of children born in January 1995, based on post-reform slope (*post-estimation*). Stepwise (rate) change is calculated as the IRD (*post-estimation*) and IRR (*main regression model*) of observed versus expected rates in January 1995.

‡Pre-reform slope corresponds to adjusted slope for fathers of children born from January 1992 to December 1994 (*main regression model*). Post-reform slope corresponds to adjusted slope for fathers of children born from January 1995 to December 1997 (*post-estimation*). Slope change is calculated as the IRR of post-reform and pre-reform slopes (*main regression model*).

IRD, incidence rate difference; IRR, incidence rate ratio; OECD, Organisation for Economic Cooperation and Development.

Sensitivity analyses are reported in online supplemental tables S1-4. Controlling for the occurrence of psychiatric hospitalisations up to 2 years before childbirth attenuated the stepwise but not slope change in post-birth psychiatric hospitalisations among migrants. Estimates for migrant subgroups revealed a stepwise *increase* among long-term residents, while others remained unchanged. Income adjustments similarly attenuated findings for migrants in general, but not subgroups. Restricting events to 18 months postpartum attenuated the stepwise but not slope change for migrants, including for non-OECD-origin migrants, and revealed stepwise increases among long-term residents. Finally, specifying the 1 January 1994 pseudo-intervention date did not affect results for natives, attenuated the stepwise change among migrants in general and revealed stepwise *increases* among non-OECD-origin, long-term and native-partnered migrant fathers, potentially reflecting residual health effects of the economic crisis.

## Discussion

This study found decreased psychiatric hospitalisation rates among migrant but not native fathers from before to after the 1995 *Father’s quota*, including for non-OECD-origin migrants and those with migrant partners. Findings for migrants in general were slightly attenuated after adjustment for pre-birth psychiatric hospitalisations and income, but decreased rates remained among migrant subgroups.

Previous quasi-experimental evidence on paid parental leave in California found no effects on fathers’ self-reported mental health.^10 30^ However, the policy introduced and promoted leave uptake to a greater degree among mothers, so the actual absence of health effects in fathers is uncertain.^31^ Similarly, although we found no changes in hospitalisation rates among Swedish-born fathers from before to after the 1995 *Father’s quota*, this does not indicate that parental leave uptake has no influence on Swedish-born fathers’ psychiatric health. Instead, given that over 60% of Swedish-born fathers used leave pre-reform, we theorise that if the pre-reform users already comprised individuals that stood to benefit the most from parental leave (ie, those in poor health or with precarious employment), a post-reform increase in uptake would not have had a sizeable impact on hospitalisation rates.

Meanwhile, we found that migrants experienced pre-reform to post-reform decreases in psychiatric hospitalisations. This may be a result of their increased leave use due to the reform’s incentives and greater awareness of their general eligibility for leave compared with before the reform. Moreover, given the cut-backs to other welfare resources during the 1990s, migrants may have been inclined to compensate for their income loss with parental leave benefits.^24^

In our subgroup analyses, we attempted to disentangle the role of integration in predicting the health gains of migrant fathers’ leave use.^16^ We found that greater duration of residence, indicative of longer receiving country exposure and greater parental leave uptake, also corresponded to greater post-reform health gains. Yet, typically ‘less integrated’ migrants, that is, those of non-OECD-origin and with foreign-born partners, experienced *greater* health gains than their OECD-origin and native-partnered peers, despite lower overall increases in uptake. This may be a result of a selective population of ‘less integrated’ migrant fathers using parental leave for the first time after the reform, including those who stood to benefit the most from parental leave (eg, due to greater work–family conflict stemming from precarious employment and occupational stressors^32^). In fact, the reform’s psychiatric benefits appeared to vary as a function of how common father’s leave was in the subgroups *before* its introduction.

We chose to examine the 1995 *Father’s quota* as it had notable effects on fathers’ parental leave uptake compared with later Swedish^14 15^ and international parental leave reforms,^10 30^ with little contamination from simultaneous parental leave policies.^33^ However, the study may have been prone to confounding by other contemporaneous factors. In 1995, there was an effort to shift the responsibility of long-term psychiatric healthcare to social services, decreasing hospitals’ capacity for severely, chronically ill psychiatric patients.^22^ We aimed to address potential bias by excluding the main deinstitutionalised group, that is, patients with schizophrenia-related diagnoses, although other unidentified diagnostic groups may have been involved. Furthermore, the study period coincided with an economic crisis which may have accelerated the deinstitutionalisation process, although with little effect on other service provision.^34^ Moreover, it may have influenced the composition of fathers (via health and socioeconomic selection into parenthood^35^) as well as created lagged psychiatric health effects, indicated by increased suicide risks.^36^ Although we attempted to account for selection bias by adjusting for pre-birth psychiatric hospitalisations and annual labour income, finding little effect on our results for Swedish-born and specific migrant groups, the significant 1994 pseudo-intervention date suggests that residual effects of the economic crisis may have remained. Future studies should consider alternate analytical methods, including using a synthetic control from different policy contexts, to remove potential bias from contemporaneous trends.^18^

Despite the rarity of the outcome, we had access to long-term national hospitalisation data from both pre-reform and post-reform periods, assuring sufficient power for our main analyses.^37^ Some subgroup analyses should be interpreted with caution. By focusing on within-group policy effects, our results were also unlikely to be affected by differential care-seeking across native and migrant subgroups.^38^ Alternate psychiatric indicators were unavailable for the study period, although our finding of changes in hospitalisations suggest that less severe psychiatric outcomes may be even more influenced by the reform.

We only had access to annual socioeconomic data, limiting our ability to assess the timing and length of fathers’ leave uptake within each calendar year. We thus excluded fathers eligible to use leave for previous children, although this impedes the study’s generalisability to all parents.^18^ The results may also have been biased by uncaptured second births during follow-up. Sensitivity analyses with 18-month follow-up, during which second births were unlikely, corroborated our main findings for migrants. Changes in migrant composition from pre-reform to post-reform were also plausible,^12^ but partly addressed through region-of-origin subgroup analyses. Finally, since we cannot account for migrants’ out-of-country experiences, our duration of residence measure excluded circular migrants with multiple immigration dates and our measure of follow-up time conservatively censored observations at the first post-birth emigration (or death and partnership dissolution).

## Conclusion

This is the first quasi-experimental study to examine the effects of a father’s leave policy on native and migrant fathers’ health. By considering specific subgroups of fathers across integration levels, this study found health effects which may have been obscured by patterns in the general population. The findings suggest that reforms intending to incentivise parental leave benefits may in fact function as an introduction to parental leave for parents with limited knowledge of the system beforehand. Furthermore, these incentives can selectively encourage disadvantaged parents, including less integrated migrants, to use leave for the first time, with corresponding benefits which can narrow social and health inequalities within the general population.

**What is already known on this subject**

- Parental leave policies are known to have protective effects for maternal and child health, but little is known about the effects on fathers’ health, including their mental health. Furthermore, no study has examined the health effects of parental leave in marginalised populations, such as migrants.

**What this study adds**

- This quasi-experimental study investigated the effects of the 1995 *Father’s quota*, a Swedish reform incentivising fathers’ leave use, on the psychiatric health of native and migrant fathers.
- The study found evidence of decreased psychiatric hospitalisations among migrant groups with low parental leave uptake pre-reform, but not among natives.
- The findings support the importance of policy incentives to promote fathers’ parental leave participation and their mental health benefits.

## Data Availability

Data may be obtained from a third party and are not publicly available.
