# Supplementary material for: Psychiatric consequences of a father’s leave policy by nativity: a quasi-experimental study in Sweden
Source: J Epidemiol Community Health. 2021 Oct 11;76(4):367–73. doi: 10.1136/jech-2021-217980 (PMC8921563; doi:10.1136/jech-2021-217980)
Supplement: Supplementary data [file jech-2021-217980supp001.pdf]

Psychiatric consequences of a fathers' leave policy by nativity: A quasi-experimental study in Sweden

SUPPLEMENTAL MATERIAL

Table S1. First-time fathers' psychiatric hospitalisation rates 0-36 months after child's birthdate, pooled by birthdate before (1992-1994) and after (1995-1997) the 1995 *Father's quota*: Sensitivity analysis adjusted for pre-birth hospitalisations

	**Sample size**	**Step-wise change**				**Slope change**	
	**n**	**IRD**	**95% CI**	**IRR**	**95% CI**	**IRR**	**95% CI**
**Swedish-born**	165,721	-0.49	-1.46-0.49	0.82	0.56-1.21	1.00	0.99-1.02
**Migrant**	32,868	-0.42	-2.18-1.35	0.89	0.65-1.23	0.97	0.95-0.98
By region of origin							
OECD	17,132	-1.28	-3.88-1.31	0.82	0.49-1.39	0.99	0.96-1.03
Non-OECD	15,736	-4.17	-9.06-0.71	0.51	0.27-0.99	0.94	0.90-0.97
By duration of residence							
< 5 years	11,727	-1.52	-5.84-2.80	0.60	0.18-2.04	0.99	0.95-1.04
≥ 5 years	12,693	5.54	-0.97-12.05	1.56	0.95-2.59	0.93	0.91-0.95
By partners' nativity							
Migrant partner	22,265	-5.18	-7.45--2.91	0.28	0.17-0.47	0.98	0.95-1.01
Swedish-born partner	10,603	5.64	-1.19-12.40	1.82	0.82-4.07	0.95	0.91-1.00

Abbreviations: CI, Confidence Interval; IRD, Incidence Rate Difference; IRR, Incidence Rate Ratio; OECD, Organisation for Economic Cooperation and Development. Rates are expressed as hospitalization events per 1,000 person-years (PY). Rate change is calculated as the incidence rate difference and incidence rate ratio of post-reform (Jan 1995) rates compared to the counterfactual (i.e., based on pre-reform trends). Slope change is calculated as the incidence rate ratio of post- and pre-reform slopes. All models are adjusted for seasonality and occurrence of psychiatric hospitalisations up to two years before childbirth (i.e., pre-birth). Confidence intervals are calculated with Huber-White-Sandwich standard errors, unless autocorrelation is present, when calculated using lag-adjusted Newey-West standard errors.

Psychiatric consequences of a fathers' leave policy by nativity: A quasi-experimental study in Sweden

Table S2. First-time fathers' psychiatric hospitalization rates 0-36 months after child's birthdate, pooled by birthdate before (1992-1994) and after (1995-1997) the 1995 *Father's quota*: Sensitivity analysis adjusted for annual labour income

	**Sample size**	**Step-wise change**				**Slope change**	
	**n**	**IRD**	**95% CI**	**IRR**	**95% CI**	**IRR**	**95% CI**
**Swedish-born**	165,721	-0.35	-1.80-1.09	0.85	0.59-1.24	1.00	0.97-1.03
**Migrant**	32,868	-3.55	-7.33-0.24	0.75	0.52-1.09	0.92	0.87-0.97
By region of origin							
OECD	17,132	-1.92	-4.72-0.88	0.80	0.49-1.30	0.97	0.92-1.02
Non-OECD	15,736	-6.96	-18.49-4.58	0.46	0.18-1.89	0.90	0.85-0.96
By duration of residence							
< 5 years	11,727	-3.90	-9.62-1.83	0.47	0.14-1.49	0.98	0.92-1.05
≥ 5 years	12,693	-0.80	-5.36-3.77	0.97	0.65-1.44	0.94	0.90-0.98
By partners' nativity							
Migrant partner	22,265	-10.70	-22.67-1.27	0.25	0.16-0.40	0.94	0.87-1.01
Swedish-born partner	10,603	1.76	-4.82-8.34	1.38	0.57-3.38	0.97	0.92-1.02

Abbreviations: CI, Confidence Interval; IRD, Incidence Rate Difference; IRR, Incidence Rate Ratio; OECD, Organisation for Economic Cooperation and Development. Rates are expressed as hospitalisation events per 1,000 person-years (PY). Rate change is calculated as the incidence rate difference and incidence rate ratio of post-reform (Jan 1995) rates compared to the counterfactual (i.e., based on pre-reform trends). Slope change is calculated as the incidence rate ratio of post- and pre-reform slopes. All models are adjusted for seasonality and fathers' annual labour income in the year before childbirth. Confidence intervals are calculated with Huber-White-Sandwich standard errors, unless autocorrelation is present, when calculated using lag-adjusted Newey-West standard errors.

Psychiatric consequences of a fathers' leave policy by nativity: A quasi-experimental study in Sweden

Table S3. First-time fathers' psychiatric hospitalisation rates 0-18 months after child's birthdate, pooled by birthdate before (1992-1994) and after (1995-1997) the 1995 *Father's quota*

	**Sample size**	**Step-wise change**				**Slope change**	
	**n**	**IRD**	**95% CI**	**IRR**	**95% CI**	**IRR**	**95% CI**
**Swedish-born**	165,721	-0.29	-0.80-0.23	0.76	0.45-1.26	1.00	0.97-1.03
**Migrant**	32,868	-0.56	-2.56-1.45	0.83	0.43-1.61	0.95	0.93-0.98
By region of origin							
OECD	17,132	-1.20	-3.02-0.62	0.63	0.33-1.19	0.98	0.95-1.01
Non-OECD	15,736	0.02	-3.81-3.86	1.00	0.33-3.01	0.92	0.86-0.98
By duration of residence							
< 5 years	11,727	-2.90	-5.63--0.17	0.18	0.05-0.60	0.99	0.94-1.04
≥ 5 years	12,693	3.60	-5.85-13.04	1.56	0.51-4.74	0.88	0.83-0.94
By partners' nativity							
Migrant partner	22,265	-1.88	-2.83--0.92	0.33	0.21-0.52	0.99	0.95-1.02
Swedish-born partner	10,603	2.16	-2.32-6.65	1.39	0.67-2.92	0.91	0.86-0.95

Abbreviations: CI, Confidence Interval; IRD, Incidence Rate Difference; IRR, Incidence Rate Ratio; OECD, Organisation for Economic Cooperation and Development. Rates are expressed as hospitalisation events per 1,000 person-years (PY). Rate change is calculated as the incidence rate difference and incidence rate ratio of post-reform (Jan 1995) rates compared to the counterfactual (i.e., based on pre-reform trends). Slope change is calculated as the incidence rate ratio of post- and pre-reform slopes. All models are adjusted for seasonality. Confidence intervals are calculated with Huber-White-Sandwich standard errors, unless autocorrelation is present, when calculated using lag-adjusted Newey-West standard errors.

Psychiatric consequences of a fathers' leave policy by nativity: A quasi-experimental study in Sweden

Table S4. First-time fathers' psychiatric hospitalisation rates 0-36 months after child's birthdate, pooled by birthdate before (1992-1994) and after (1995-1997) the 1995 *Father's quota*: Sensitivity analysis with 1994 pseudo-intervention date

	**Sample size**	**Step-wise change**				**Slope change**	
	**n**	**IRD**	**95% CI**	**IRR**	**95% CI**	**IRR**	**95% CI**
**Swedish-born**	165,721	-0.18	-1.25-0.99	0.94	0.62-1.43	1.00	0.97-1.03
**Migrant**	32,868	-0.45	-3.32-2.43	0.93	0.59-1.46	0.95	0.93-0.98
By region of origin							
OECD	17,132	-2.61	-6.07-0.86	0.67	0.41-1.12	0.96	0.93-0.99
Non-OECD	15,736	2.81	-1.40-7.02	1.60	0.79-3.23	0.95	0.91-0.99
By duration of residence							
< 5 years	11,727	-5.18	-12.39-2.04	0.40	0.14-1.17	0.94	0.89-0.99
≥ 5 years	12,693	5.07	-9.18-19.33	1.59	0.36-6.98	0.94	0.86-1.02
By partners' nativity							
Migrant partner	22,265	-1.17	-5.08-2.74	0.81	0.40-1.64	0.95	0.92-0.99
Swedish-born partner	10,603	2.78	-4.71-10.26	1.39	0.58-3.35	0.95	0.89-1.01

Abbreviations: CI, Confidence Interval; IRD, Incidence Rate Difference; IRR, Incidence Rate Ratio; OECD, Organisation for Economic Cooperation and Development. Rates are expressed as hospitalisation events per 1,000 person-years (PY). Rate change is calculated as the incidence rate difference and incidence rate ratio of post-reform (Jan 1994) rates compared to the counterfactual (i.e., based on pre-reform trends). Slope change is calculated as the incidence rate ratio of post- and pre-reform slopes. All models are adjusted for seasonality. Confidence intervals are calculated with Huber-White-Sandwich standard errors, unless autocorrelation is present, when calculated using lag-adjusted Newey-West standard errors.

Psychiatric consequences of a fathers' leave policy by nativity: A quasi-experimental study in Sweden

Figure S1. Fathers' mean age at childbirth (years) before (1992-1994) and after (1995-1997) the 1995 *Father's quota* (by child's date of birth): Swedish-born and migrant fathers

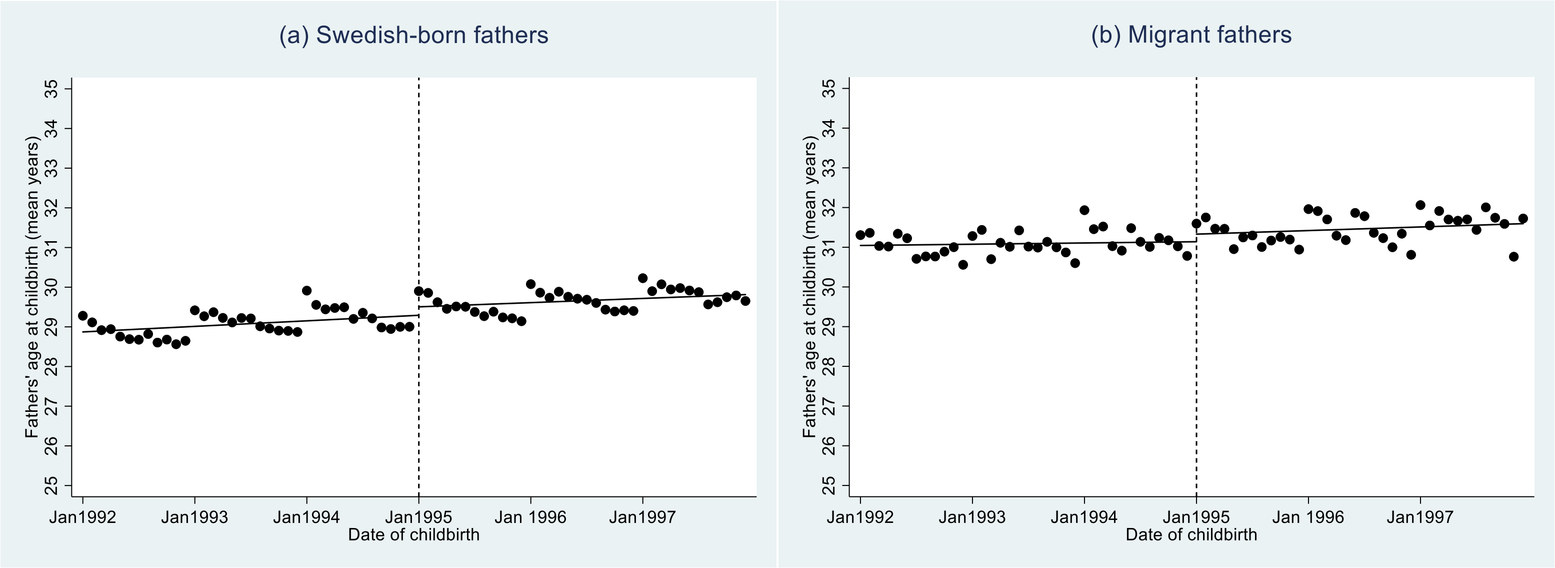

Psychiatric consequences of a fathers' leave policy by nativity: A quasi-experimental study in Sweden

Figure S2. Fathers' mean annual labour income (in thousands of Swedish Kronor) before (1992-1994) and after (1995-1997) the 1995 *Father's quota* (by child's date of birth): Swedish-born and migrant fathers

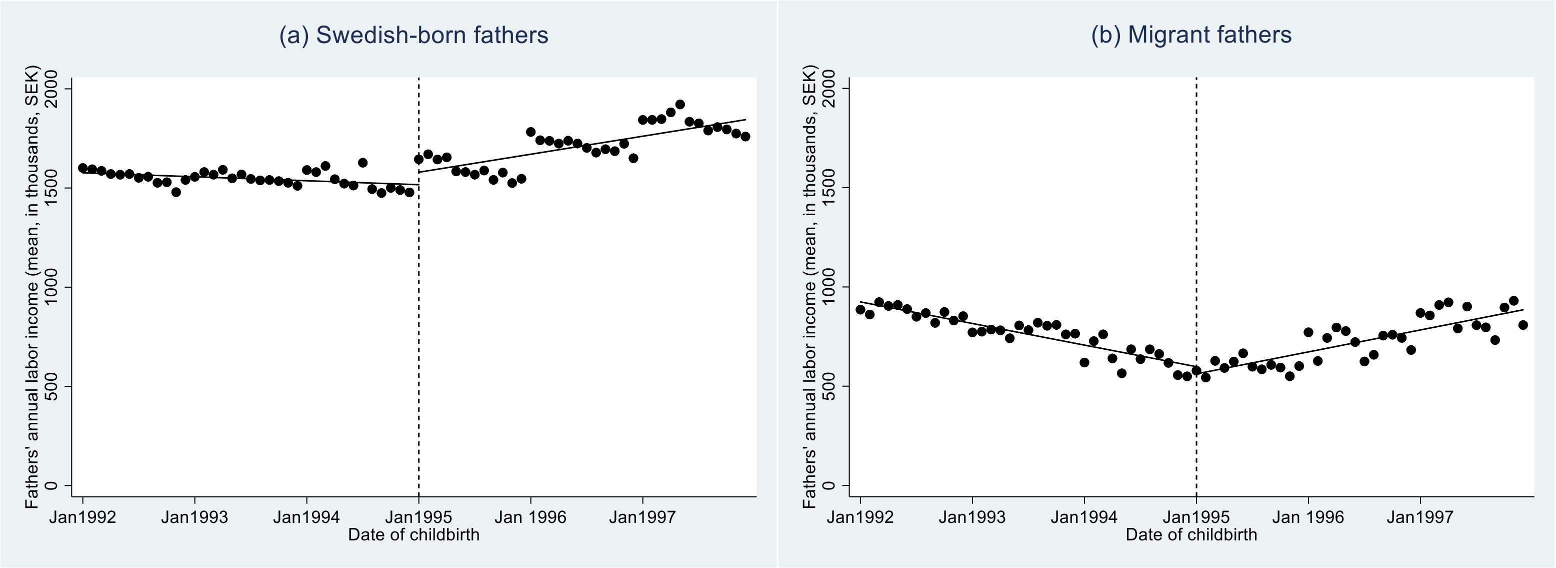

Psychiatric consequences of a fathers' leave policy by nativity: A quasi-experimental study in Sweden

Figure S3. Migrant fathers' characteristics before (1992-1994) and after (1995-1997) the 1995 *Father's quota* (by child's date of birth): By region of origin and partners' nativity

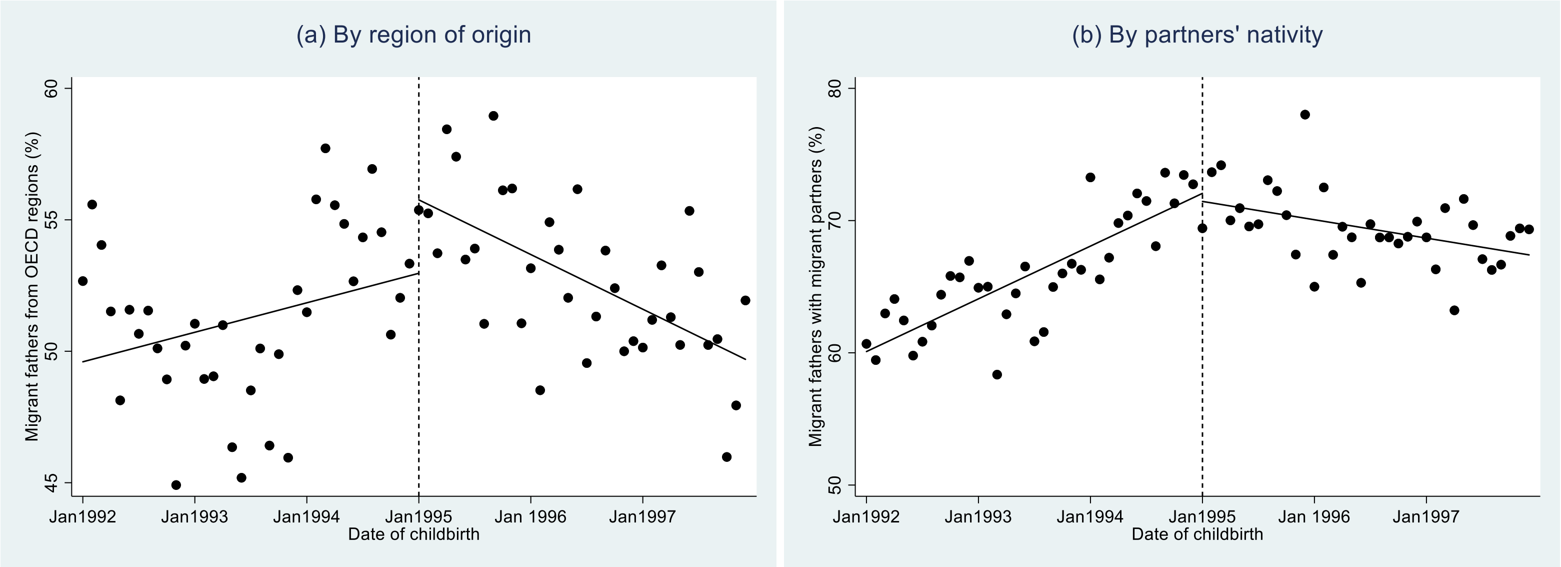

We chose not to plot duration of residence given the large proportion of missing data (i.e., for individuals with multiple migrations).

Psychiatric consequences of a fathers' leave policy by nativity: A quasi-experimental study in Sweden

Figure S4. Proportion of fathers with pre-birth hospitalisations with a mental health diagnosis (up to two years prior to birth), before (1992-1994) and after (1995-1997) the 1995 *Father's quota* (by child's date of birth): Swedish-born and migrant fathers

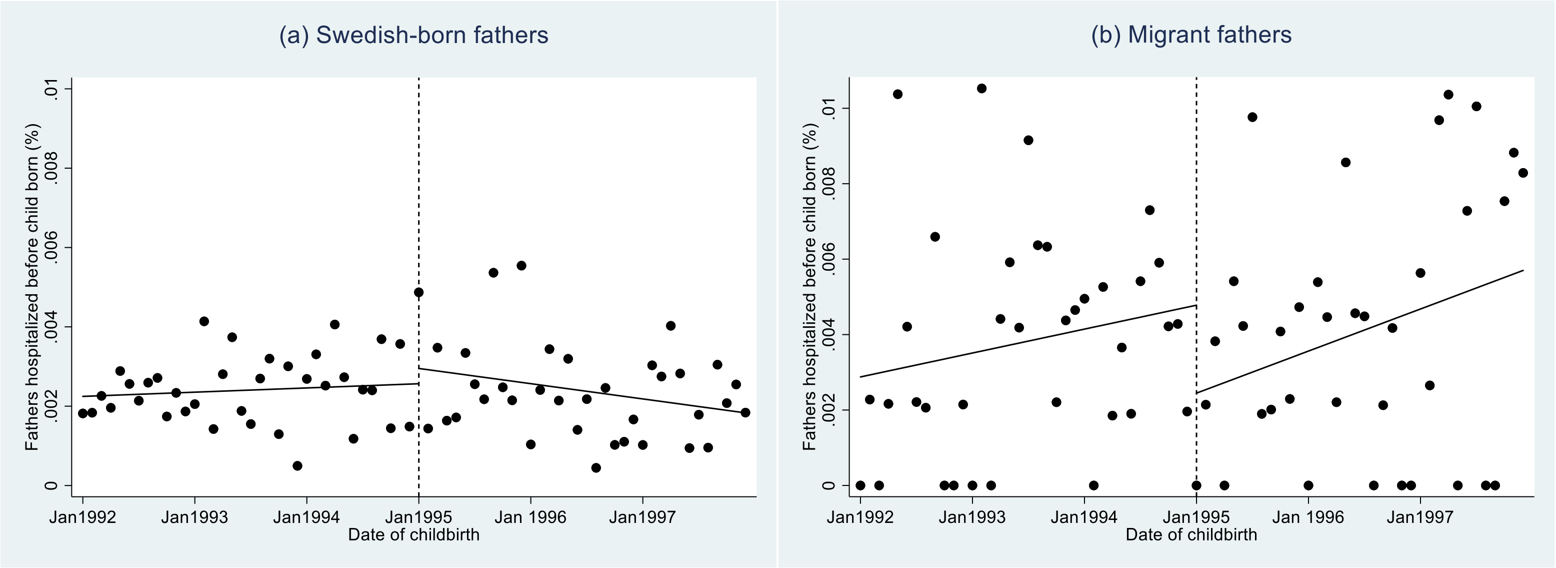

Psychiatric consequences of a fathers' leave policy by nativity: A quasi-experimental study in Sweden

Figure S5. First-time fathers' parental leave use before (1992-1994) and after (1995-1997) the 1995 *Father's quota* (by child's date of birth): Swedish-born and migrant fathers

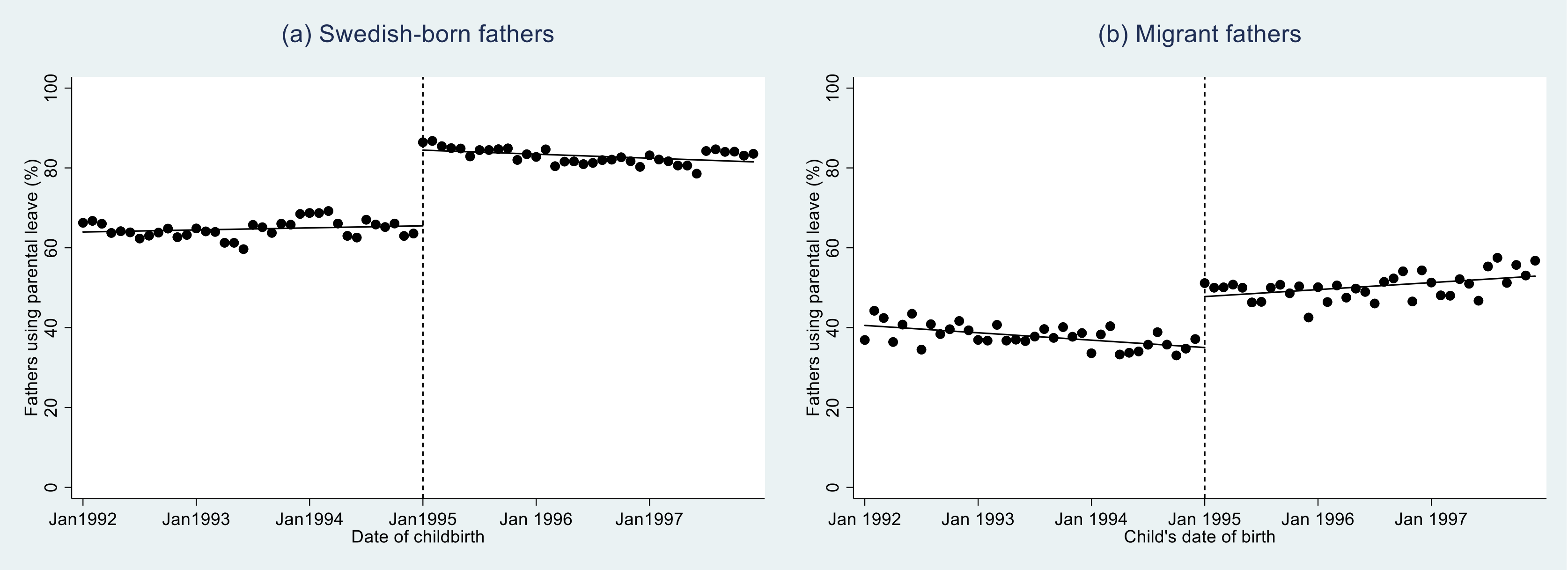

Psychiatric consequences of a fathers' leave policy by nativity: A quasi-experimental study in Sweden

Figure S6. First-time fathers' parental leave use before (1992-1994) and after (1995-1997) the 1995 *Father's quota* (by child's date of birth): OECD- and non-OECD-origin migrant fathers

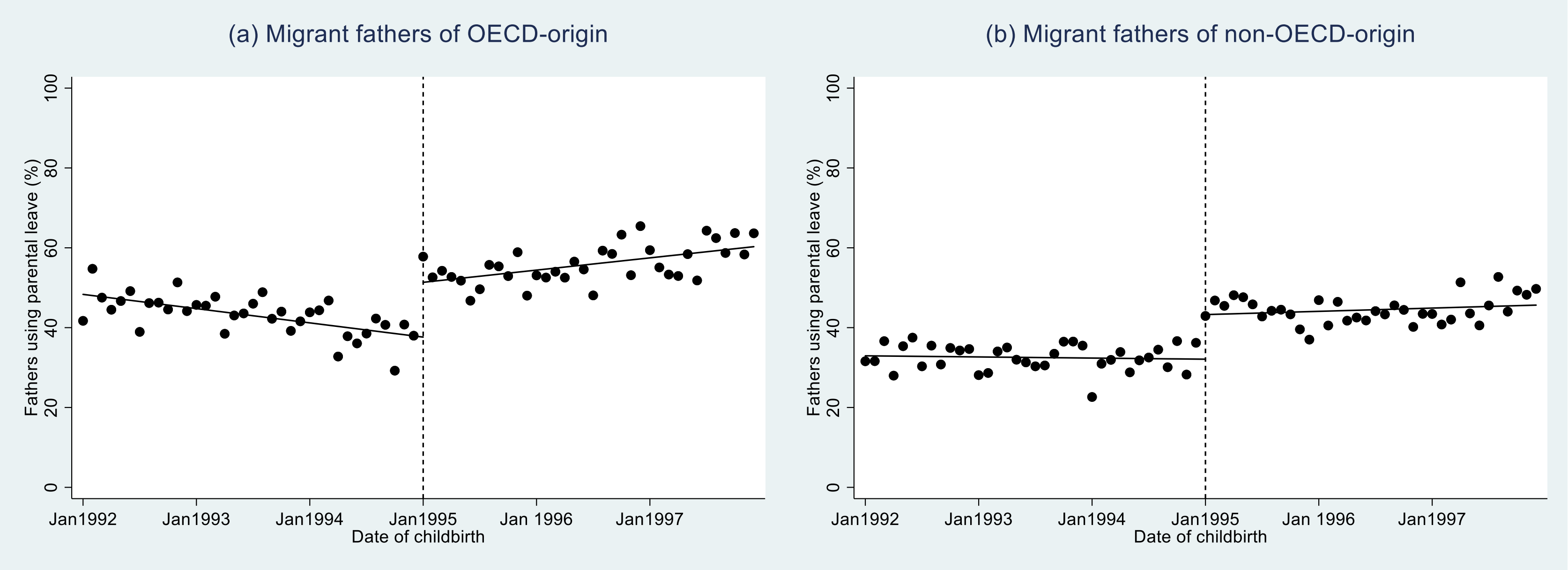

Psychiatric consequences of a fathers' leave policy by nativity: A quasi-experimental study in Sweden

Figure S7. First-time fathers' parental leave use before (1992-1994) and after (1995-1997) the 1995 *Father's quota* (by child's date of birth): Migrant fathers residing in Sweden <5 and ≥5 years

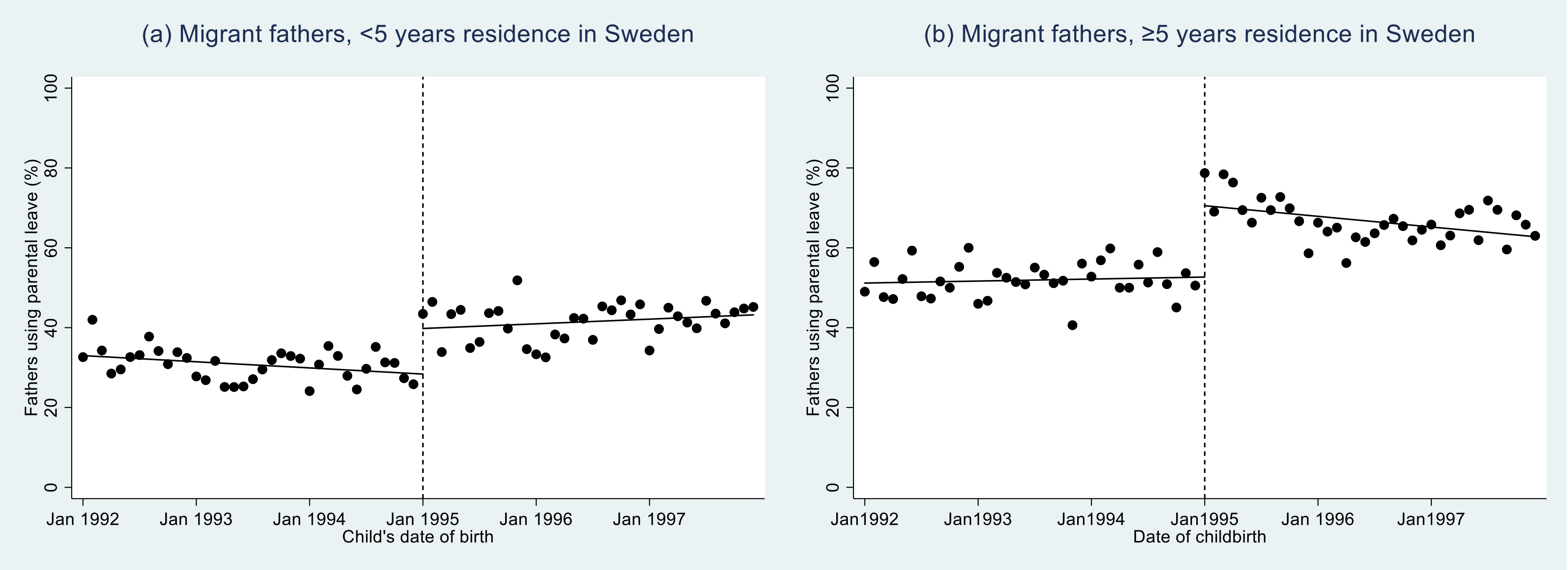

Psychiatric consequences of a fathers' leave policy by nativity: A quasi-experimental study in Sweden

Figure S8. First-time fathers' parental leave use before (1992-1994) and after (1995-1997) the 1995 *Father's quota* (by child's date of birth): Migrant fathers with migrant partners and Swedish-born partners

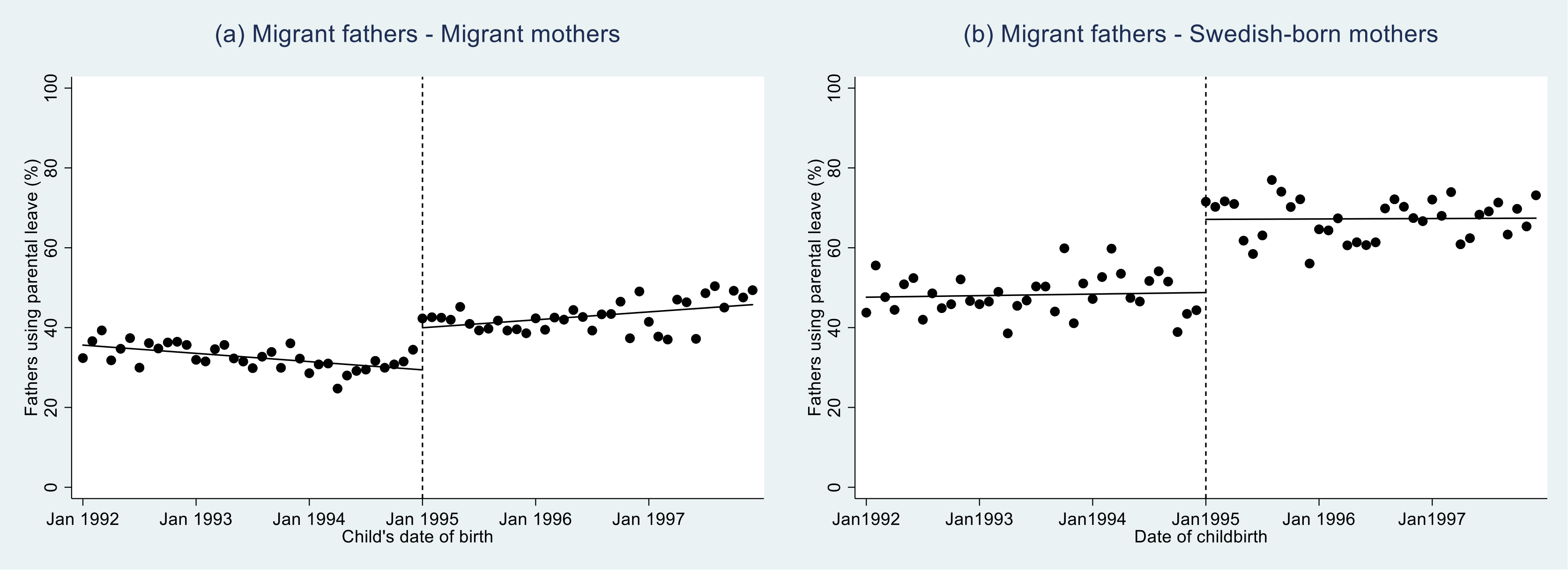

Psychiatric consequences of a fathers' leave policy by nativity: A quasi-experimental study in Sweden

Figure S9. Monthly time-series plots of first-time fathers' psychiatric hospitalisation rates 0-36 months after child's birthdate, pooled by child's birth month (Jan 1992-Dec 1997), interrupted by *Father's quota* (Jan 1995). A) Migrant fathers of OECD origin (n=17,132). B) Migrant fathers of non-OECD origin (n=15,736).

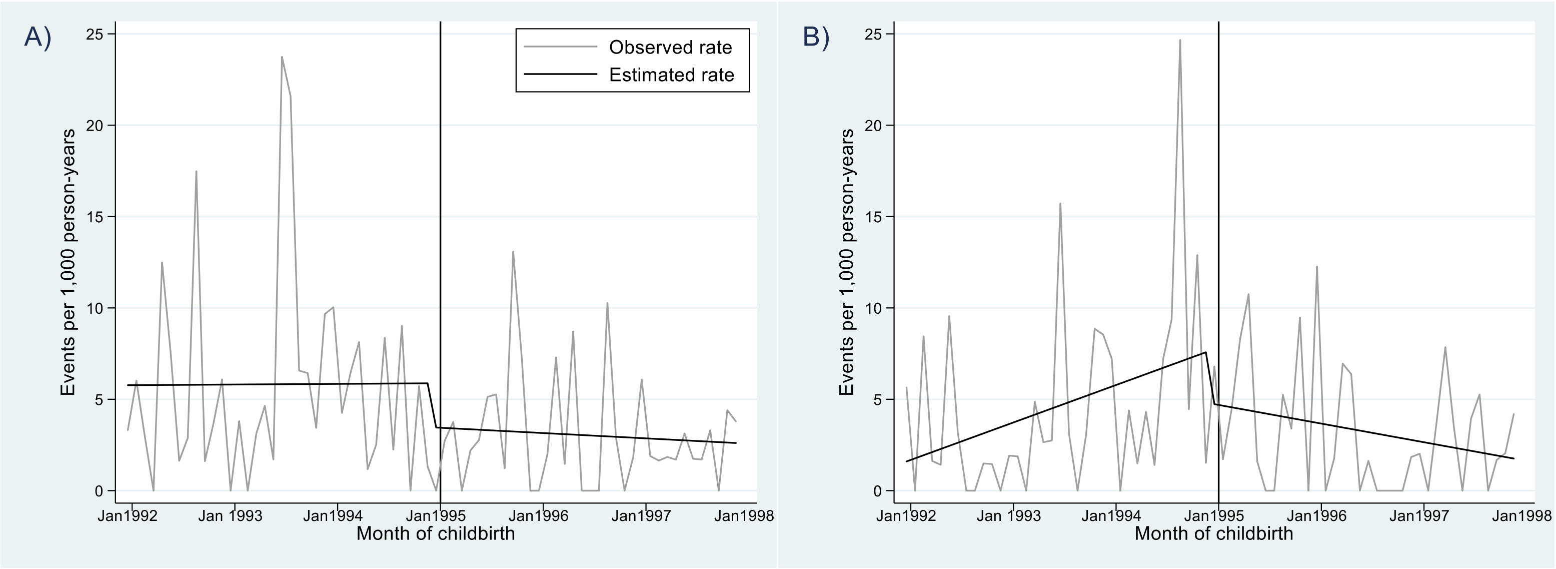

Observed rate is the unadjusted hospitalisation rate of fathers by child's birthdate (monthly data), pooled across the first 36 months after birth. Estimated rate is the adjusted average hospitalisation rate estimated from the fully-adjusted negative binomial regression model with twelve-month moving average filters to de-seasonalise the rate.

Psychiatric consequences of a fathers' leave policy by nativity: A quasi-experimental study in Sweden

Figure S10. Monthly time-series plots of first-time fathers' psychiatric hospitalisation rates 0-36 months after child's birthdate, pooled by child's birth month (Jan 1992-Dec 1997), interrupted by *Father's quota* (Jan 1995). A) Migrant fathers, resident <5 years in Sweden (n=11,727). B) Migrant fathers, resident ≥5 years in Sweden (n=12,693).

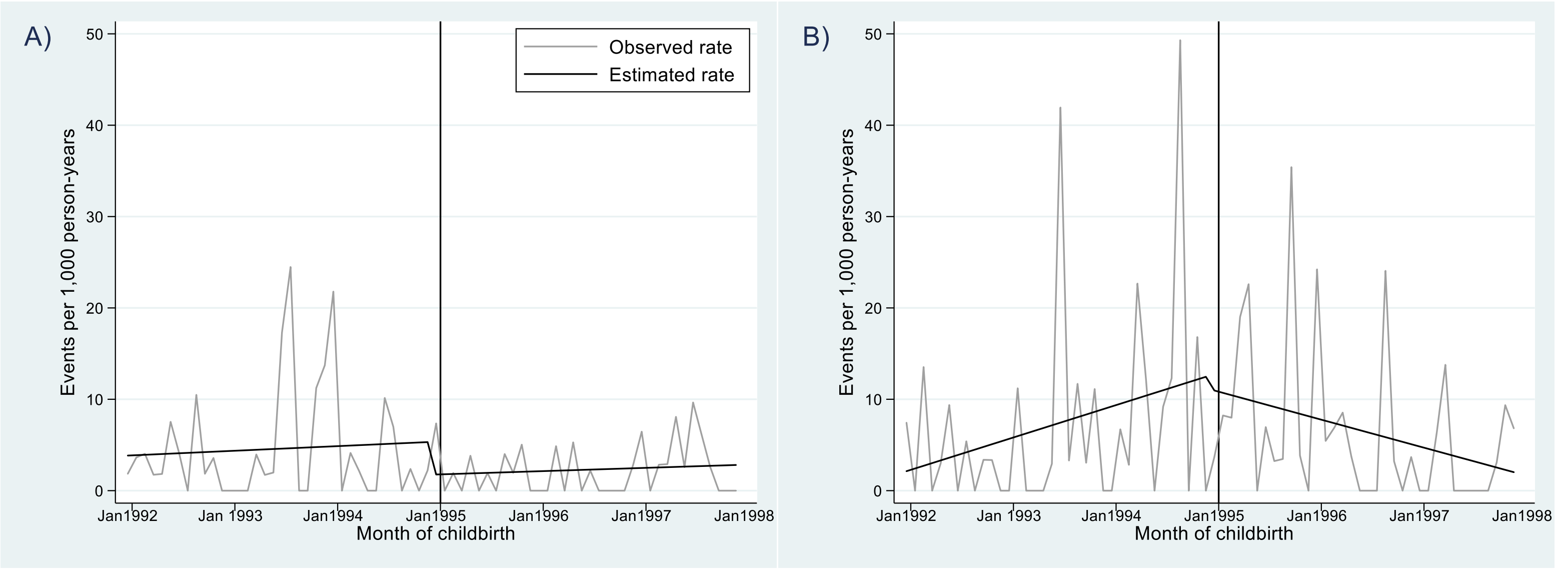

Observed rate is the unadjusted hospitalisation rate of fathers by child's birthdate (monthly data), pooled across the first 36 months after birth. Estimated rate is the adjusted average hospitalisation rate estimated from the fully-adjusted negative binomial regression model with twelve-month moving average filters to de-seasonalise the rate.

Psychiatric consequences of a fathers' leave policy by nativity: A quasi-experimental study in Sweden

Figure S11. Monthly time-series plots of first-time fathers' psychiatric hospitalisation rates 0-36 months after child's birthdate, pooled by child's birth month (Jan 1992-Dec 1997), interrupted by *Father's quota* (Jan 1995). A) Migrant fathers – Migrant mothers (n=22,265). B) Migrant fathers – Swedish-born mothers (n=10,603).

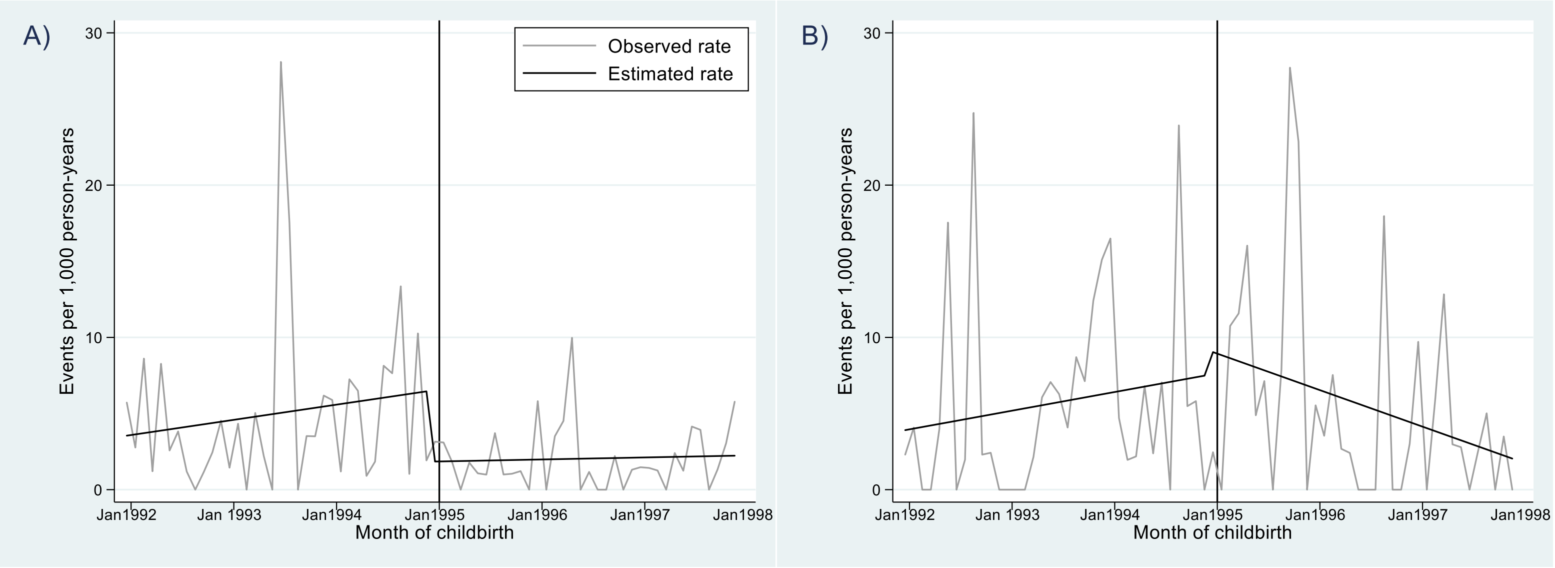

Observed rate is the unadjusted hospitalisation rate of fathers by child's birthdate (monthly data), pooled across the first 36 months after birth. Estimated rate is the adjusted average hospitalisation rate estimated from the fully-adjusted negative binomial regression model with twelve-month moving average filters to de-seasonalise the rate.
